# A novel c.64G > T (p.G22C) *NR5A1* variant in a Chinese adolescent with 46,XY disorders of sex development: a case report

**DOI:** 10.1186/s12887-023-03974-7

**Published:** 2023-04-19

**Authors:** Dan Zhang, Dajia Wang, Yajie Tong, Mingyu Li, Lingzhe Meng, Qiutong Song, Ying Xin

**Affiliations:** 1 Department of Pediatrics, Shengjing Hospital of China Medical University, Shenyang, Liaoning, 110004 People’s Republic of China; 2 Department of Clinical Laboratory, Shengjing Hospital of China Medical University, Shenyang, Liaoning, 110004 People’s Republic of China

**Keywords:** 46,XY disorders of sex development, NR5A1, Anti-Müllerian hormone, Adolescent

## Abstract

**Background:**

Adolescents with 46,XY disorders of sex development (DSD) face additional medical and psychological challenges. To optimize management and minimize hazards, correct and early clinical and molecular diagnosis is necessary.

**Case presentation:**

We report a 13-year-old Chinese adolescent with absent Müllerian derivatives and suspected testis in the inguinal area. History, examinations, and assistant examinations were available for clinical diagnosis of 46,XY DSD. The subsequent targeting specific disease‐causing genes, comprising 360 endocrine disease-causing genes, was employed for molecular diagnosis. A novel variation in nuclear receptor subfamily 5 group A member 1 (*NR5A1*) [c.64G > T (p.G22C)] was identified in the patient. In vitro functional analyses of the novel variant suggested no impairment to *NR5A1* mRNA or protein expression relative to wild-type, and immunofluorescence confirmed similar localization of NR5A1 mutant to the cell nucleus. However, we observed decreased DNA-binding affinity by the *NR5A1* variant, while dual-luciferase reporter assays showed that the mutant effectively downregulated the transactivation capacity of anti-Müllerian hormone. We described a novel *NR5A1* variant and demonstrated its adverse effects on the functional integrity of the NR5A1 protein resulting in serious impairment of its modulation of gonadal development.

**Conclusions:**

This study adds one novel *NR5A1* variant to the pool of pathogenic variants and enriches the adolescents of information available about the mutation spectrum of this gene in Chinese population.

**Supplementary Information:**

The online version contains supplementary material available at 10.1186/s12887-023-03974-7.

## Background

46,XY disorders/differences of sex development (DSD) occur with a frequency of approximately 1:20,000 [1] and encompass complete or partial gonadal dysgenesis, undervirilisation or under-masculinisation of an XY male due to genetic variation, abnormal hormone secretion, or abnormal changes in peripheral sensitivity to testosterone [2, 3]. A great proportion of 46,XY is caused by mutations in key transcription factors required for sex differentiation and androgen biosynthesis or action [2]. Compared with other age stages, adolescents with 46,XY DSD face additional medical and psychological challenges, which are particularly prominent and difficult for newly diagnosed adolescents [3]. To optimize management and minimize hazards, correct and timely diagnosis is necessary [3]; however, the diagnosis rate of 46,XY DSD with gonadal hypoplasia is exceptionally low [4], with > 50% of patients not receiving a molecular diagnosis [5, 6]. Clinical, biochemical, and imaging tests are recommended as the initial method for all suspected DSD patients. The classic diagnostic approach emphasizes obtaining these assessments before conducting genetic analyses (often limited to individual candidate genes) and is expensive, laborious, and time-consuming. The new approach proposes genetic testing as the first-line investigation after karyotyping and selective subsequent investigation to detail the phenotype [7, 8]. We implemented both approaches in parallel in the diagnosis of a Chinese adolescent with 46,XY DSD. The diagnostic process was extensive. According to the medical history, physical examinations, karyotyping, gonadotropin levels test, and ultrasound examinations, it was not difficult to obtain clinical diagnosis as 46,XY DSD. The subsequent genetic sequencing provided with the molecular diagnosis as a novel variant of *NR5A1* c.64G > T (p.G22C).

Nuclear receptor subfamily 5 group A member 1 (*NR5A1,* also known as *SF-1*, *AD4BP* and *FTZF1*) is a key transcription factor that determines gonadal development and regulates coordinates endocrine functions [9]. *NR5A1* variants occur in ~ 15–20% of patients and are regarded as a common genetic pathogeny of 46,XY DSD [5, 10, 11]. Achermann et al*.* [12] described the first two *NR5A1* variants in individuals with 46,XY DSD who presented primary adrenal insufficiency and complete gonadal dysgenesis. Additionally, Pedace et al*.* [13] reviewed 61 *NR5A1* variants among 81 cases with 46,XY DSD in 2014, while Fabbri-Scallet et al*.* [6] reported an update of 188 variants of *NR5A1* in 238 cases with 46,XY DSD in 2020. These studies revealed that pathogenic variants are reported in 35–45% of individuals with 46,XY DSD [8, 11, 14]; however, there were very few cases diagnosed in adolescence. In addition, the evidence supported by experimental data is imperative to determine the role of these variants in 46,XY DSD [2]. Here, we describe the diagnostic process of a Chinese adolescent with 46,XY DSD and share our experience to bringing more attention to adolescents with 46,XY DSD. To the best of our knowledge, there has been no report of the c.64G > T (p.G22C) *NR5A1* variant; in vitro functional study of this novel variant will expand our knowledge of the *NR5A1* mutational spectrum.

## Case presentation

**Fig. 1:**
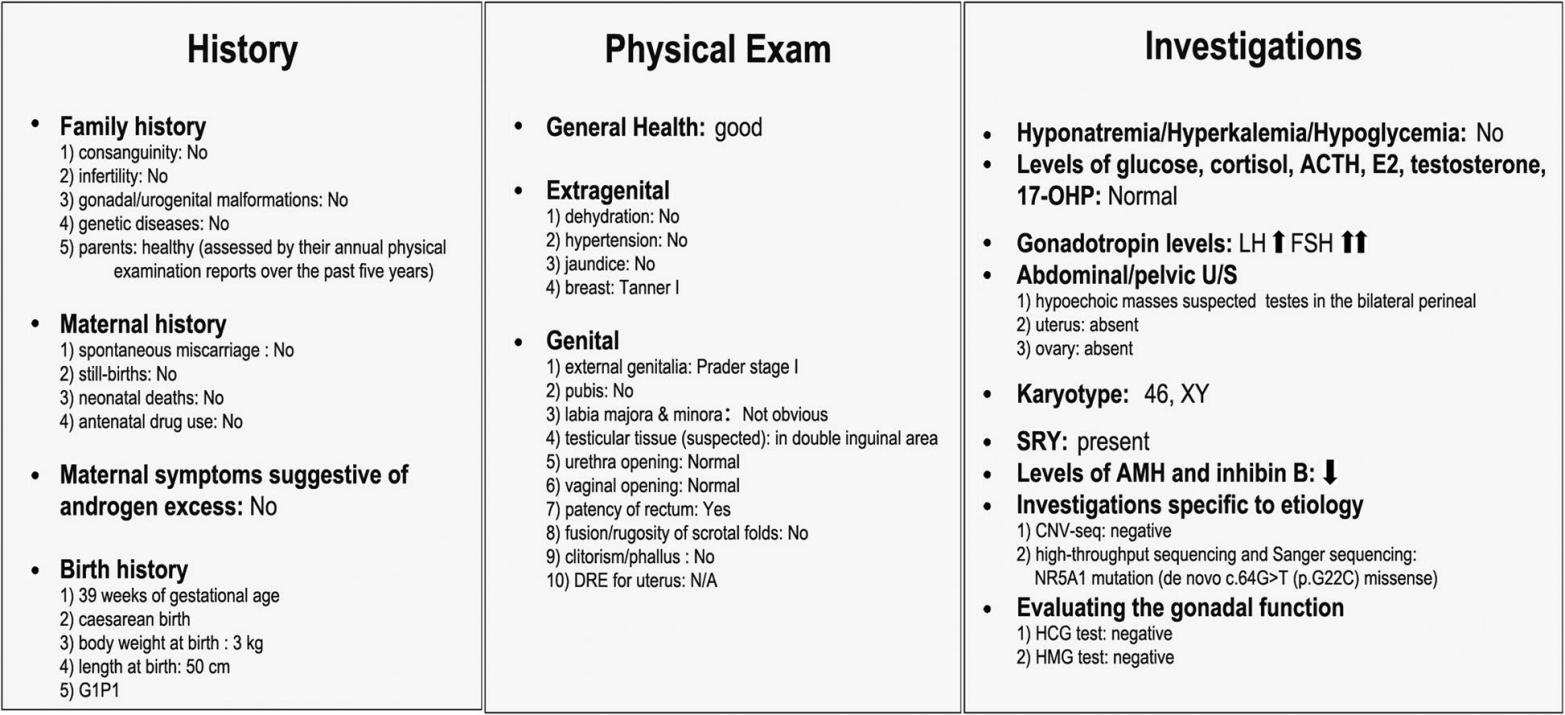
Summary of data related to the diagnosis of an adolescent with 46,XY DSD

The adolescent was 13-years-old female initially treated for right lower abdominal pain. When the patient came back to the hospital for a check, the ultrasound examination showed absent Mullerian derivatives and suspected testis in the inguinal area. Since then, it has opened a complicated visit for nearly 10 months (Supplementary Fig. 1). The patient is the only child of healthy nonconsanguineous parents and was born through caesarean birth at 39 weeks of gestational age. The adolescent presented with breast in Tanner stage 1, undeveloped female external genitalia and suspected testicular tissue in double inguinal area. The External Genital Score (EGS) of the patient was 1, indicating that the patient's external genitalia is closer to that of female. The patient did not have symptoms or signs of adrenal insufficiency. The laboratory examinations (Table 1) suggested hypergonadotropic hypogonadism, 46,XY karyotype, and present *SRY* gene. The levels of AMH and inhibin B in this patient, evaluated by referring to the reference values in the literatures [15, 16], were significantly decreased. These clinical data (Fig. 1) supported the diagnosis as 46,XY DSD.

**Table 1:**
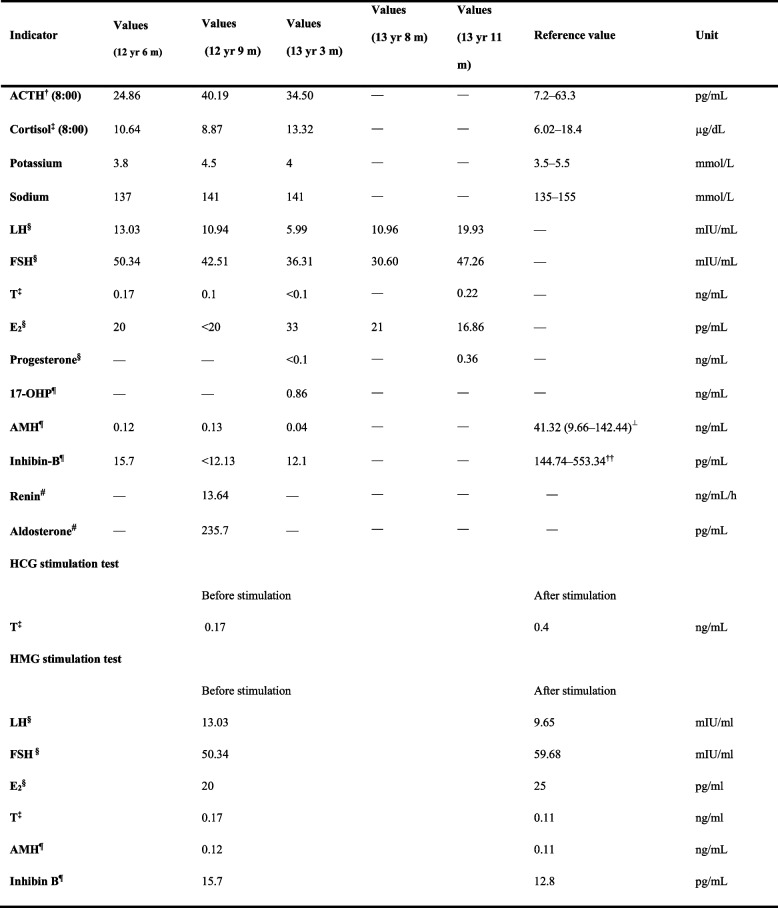
The laboratory examinations of the adolescent at diagnosis and follow-up visits

*ACTH* Adrenocorticotropic hormone, *LH* Luteinizing hormone, *FSH* Follicle-stimulating hormone, *T* Testosterone, *E2* Estradiol, *17-OHP* 17-hydroxyprogesterone, *HCG*, Human chorionic gonadotropin, HMG, human menopausal gonadotropin

^†^Captured by luminescence, ‡Captured by chemiluminescence, **§**Captured by electrochemiluminescence, Captured using ELISA, **#** Renin (recumbent position) and aldosterone (recumbent position), ⊥Reference serum AMH levels in normal boys at 12.5 years old (Median (3rd–97th percentiles)) in Reference 15, ††Reference serum Inhibin-B levels in normal boys at 12 years old in Reference 16

**Fig. 2:**
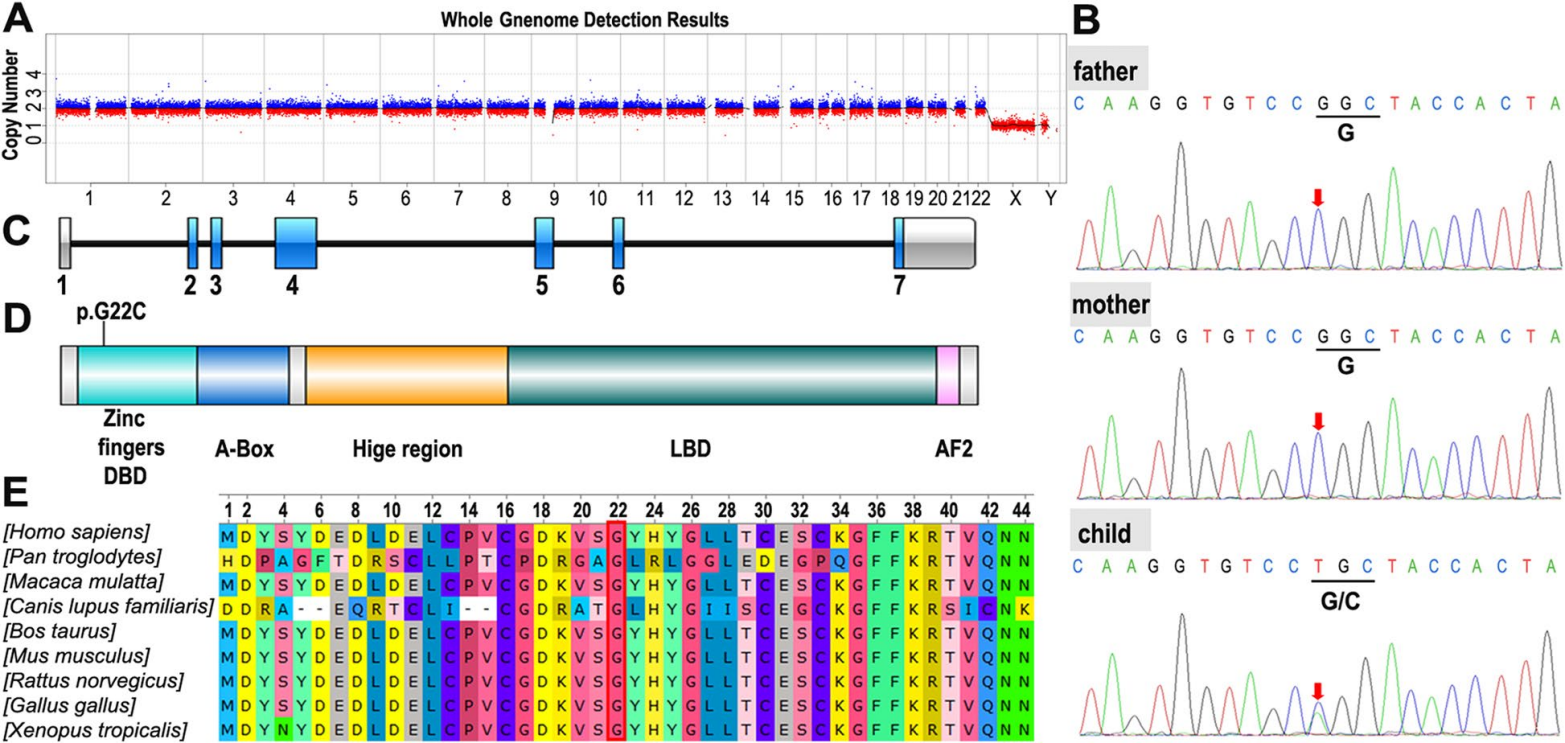
The sequencing results and bioinformatics analysis of mutation site. **A** CNV-seq analysis results showed the identification of 46,XY with no chromosome aneuploidy and genome copy number variation > 100 kb. **B** Sanger sequencing results for the *NR5A1* mutation site in the patient and parents. The red arrow shows the mutation site. **C** Schematic representation of the c.64 G > T variant located in exon 2 of *NR5A1*. **D** Schematic representation of the G22C substitution in the NR5A1 DBD. **E** Conservation of Gly22 across species

**Fig. 3:**
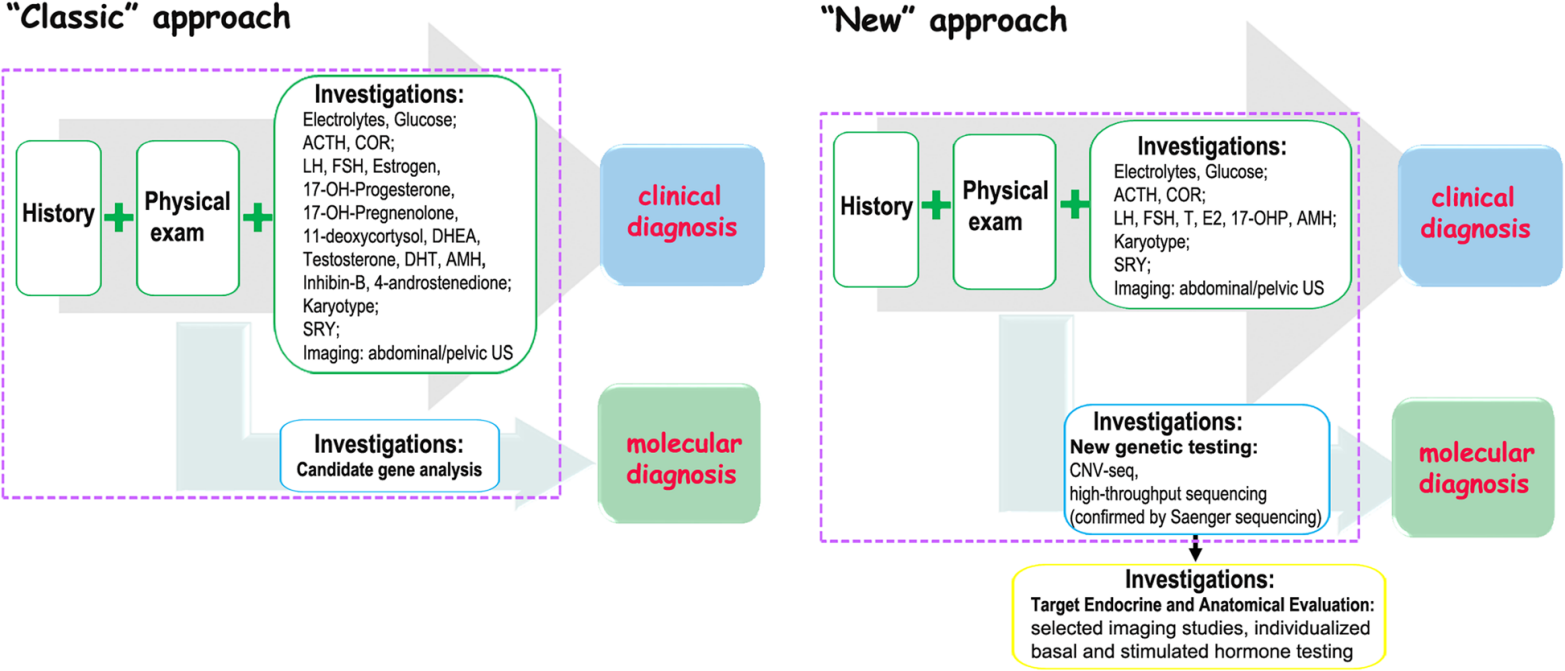
The diagnostic approaches for the diagnosis of 46,XY DSD. The “classic” and “new” approaches for the diagnosis of 46,XY DSD are summarized in the left and right panels, respectively. The purple dotted line boxes indicate the first-line information for diagnosis. We implemented both approaches in parallel during the diagnostic process of 46,XY DSD in this study

To obtain the molecular diagnosis, the subsequent genetic sequencing was performed. As chromosome microdeletion and microduplication are pathogenic factors associated with DSD, the copy number variation sequencing (CNV-seq) was performed firstly and the results indicated that there was no pathogenic variation in the patient at the chromosome level (Fig. 2A). Secondly, the targeting specific disease‐causing genes (TRS) was implemented as previously reported [17]. In brief, the exons and adjacent intron regions (± 50 bp) of 360 endocrine disease-causing genes (including DSD-causing genes; Supplementary Table 1) were captured. The sequencing results were analyzed using the related software and then a *NR5A1* variant the candidate variation was confirmed by Sanger sequencing from the adolescent and parental samples. Pathogenicity analysis identified a de novo c.64G > T (p.G22C) heterozygous and missense variant in *NR5A1* that was absent in the parents (Fig. 2B). This novel variant replaced guanine with thymine at nucleotide 64 in exon 2(Fig. 2C), leading to a glycine (Gly)-to-cysteine (Cys) substitution at position 22 in the DNA-binding domain (DBD) (Fig. 2D). Gly22 resides is highly conserved in different species (Fig. 2E). Additionally, function prediction identified this variation in *NR5A1* as harmful (Supplementary Table 2). Therefore, these findings suggested the novel c.64G > T (p.G22C) variation in *NR5A1* as the genetic cause of 46,XY DSD. In this present study, we implemented both the classic and new approach in parallel for the diagnosis of 46,XY DSD in an adolescent resulting from a c.64G > T (p.G22C) *NR5A1* variant. The diagnostic process is shown in Fig. 3.

Following the molecular diagnosis, further investigations are warranted to determine a more accurate phenotype. The three-day human chorionic gonadotropin (hCG) stimulation test and seven-day human menopausal gonadotropin (HMG) stimulation test were performed to further assess gonad function. Negative results (Table 1) indicated that both testicular endocrine function and ovarian endocrine function were poor.

**Fig. 4:**
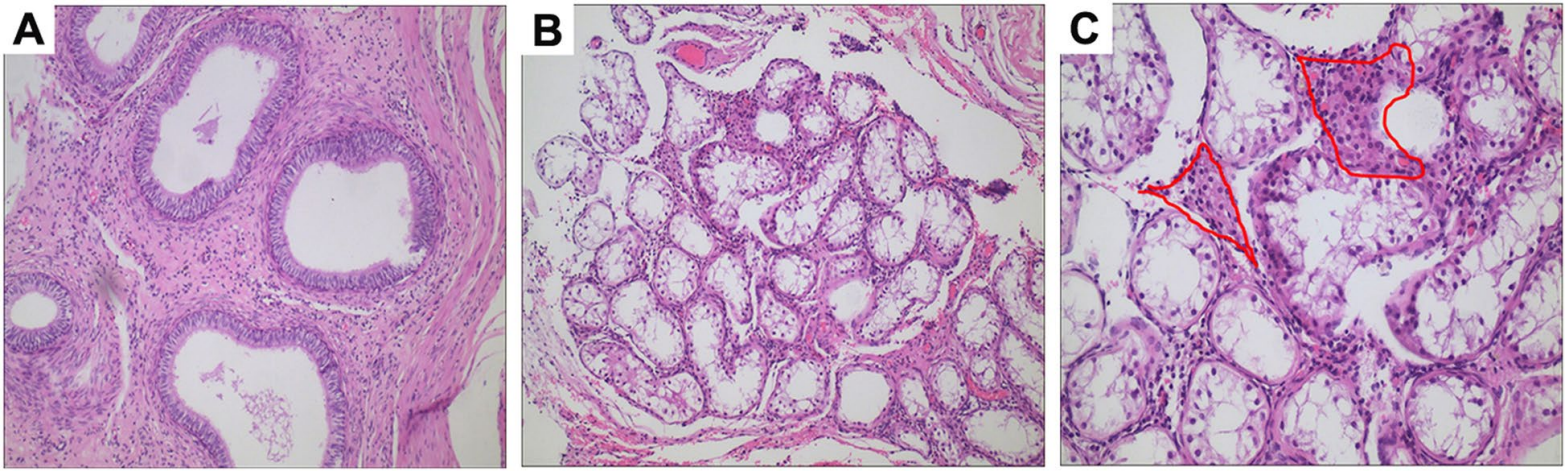
The micrograph of gonad histology of the patient (hematoxylin and eosin staining). **A** Epididymal structure showing the epididymal duct surrounded by pseudostratified columnar epithelium. The cavity surface is flat, and circular smooth muscle fibers are observed outside of the epithelium (Magnification: 100 ×). **B**&**C** Spermatogenic tubules and interstitial cells can be seen in testicular tissue. There are only Sertoli cells in seminiferous tubules rather than definite spermatogenic cells. Small clusters of interstitial cells were observed among seminiferous tubules (the areas are outlined by the red lines in Fig. C). (Magnification: B, 100 × ; C, 200 ×)

Subsequent issues involved treatment and gender assignment/reassignment. Psychological assessment revealed that the gender identity of the patient was female, which coincided with the social gender. The multi-disciplinary treatment team of DSD in our hospital conducted extensive and in-depth counseling with the adolescent and parents. Following the family agreeing to surgery, exploration results revealed absent Müllerian derivatives with blind vagina and poorly developed testicular tissue in the inguinal region without spermatogenic cells, which were confirmed by intraoperative frozen pathology and postoperative microscopic biopsy (Fig. 4). With the parents’ full knowledge and consent, the patient underwent bilateral gonadectomy. The surgical findings suggested that the patient had partial gonadal dysgenesis, which agreed with the clinical data. After careful consideration, the final decision of the family was that the gender of the adolescent to remain female.

The patient returned to our department for estrogen-replacement therapy (ERT) nearly 4 months after the surgery. We performed selective examinations of the patient at age 13 (Table 1), which revealed the following: height of 161 cm (75^th^ to 90^th^ percentile of height for girls of the same age and the same ethnic group); weight of 64.3 kg (> 90^th^ centile); BMI of 24.8; bone age of 12.5-years old. The blood pressure was normal at 111/73 mmHg. The patient was treated with a low dose of oral progynova (250 μg; Bayer Vital GmbH, Leverkusen, Germany). The breasts developed to Tanner stage 2, and estrogen levels increased after ERT. The patient received regular follow-up and ERT for > 9 months with no adverse drug reactions.

Like this case, the novel c.64G > T (p.G22C) variation in *NR5A1* also aroused our interest. The differences in the structural conformations of the WT and NR5A1-Mut variants were evaluated using the NR5A1 structure (Protein Data Bank ID: 4QJR). Despite the G22C substitution, both WT Gly22 and NR5A1-Mut Cys22 form hydrogen bonds with threonine at position 29 (Thr29) (Supplementary Fig. 2). According to the root-mean-square deviation between the WT and NR5A1-Mut (0.126), the results suggested that the G22C variant results in minimal alteration of the three-dimensional conformation of NR5A1.

**Fig. 5:**
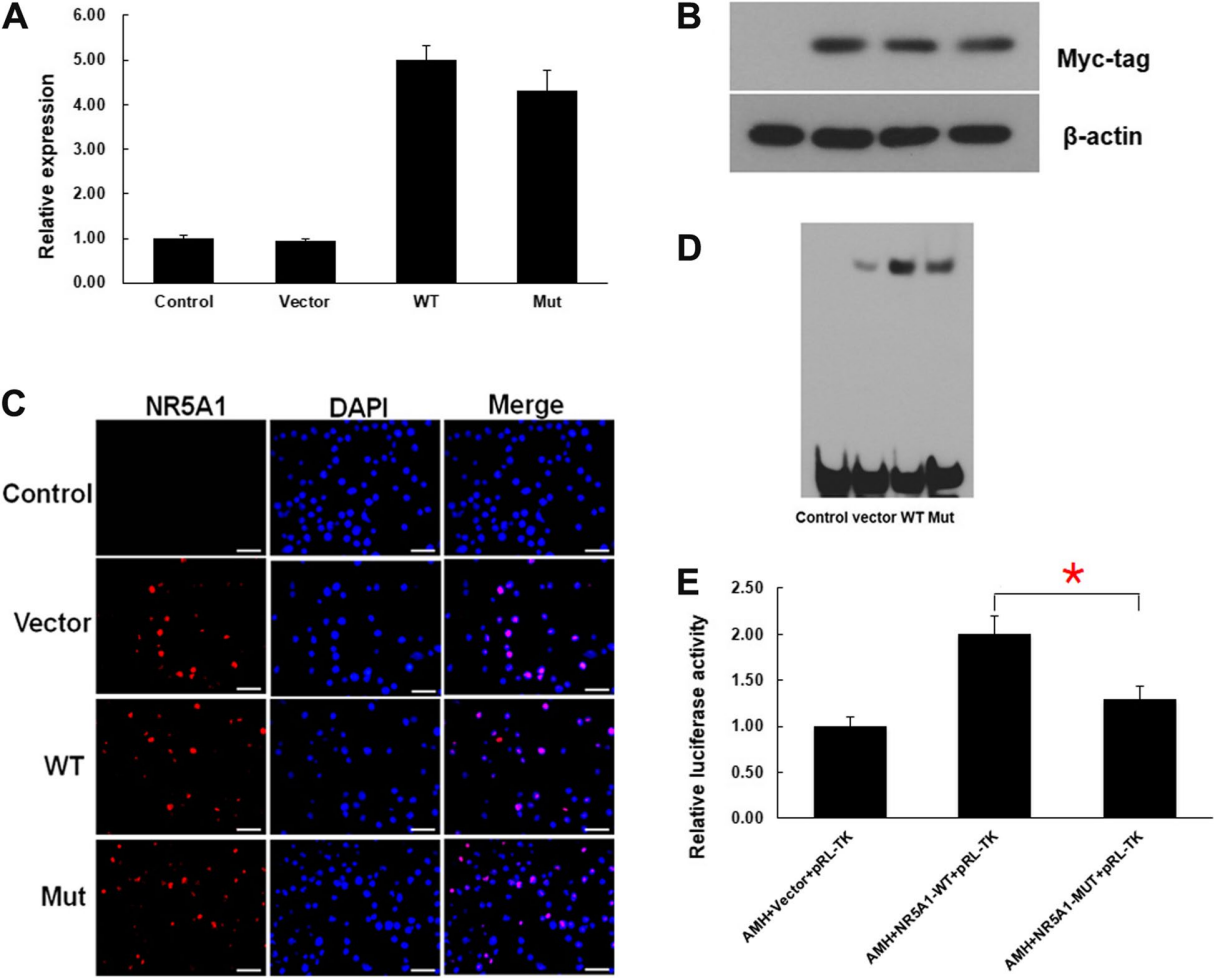
Functional analyses of the NR5A1 mutant. **A** *NR5A1* mRNA levels in 293 T cells according to qRT-PCR analysis: non-transfected (Control) and transfected with an empty vector (Vector), Myc-tagged WT (WT), or c.64G > T (p.G22C) NR5A1-Mut (Mut) vectors. **B** NR5A1 expression in 293 T cells according to western blot analysis (same grouping as **A**, the raw figures in Supplementary Fig. 3A&B.). **C** Nuclear localization of the NR5A1 mutant according to immunocytochemical analysis (same grouping as **A**). Scale bar, 50 μm. **D** EMSAs results showing altered DNA binding by the NR5A1 mutant. Nuclear extracts were prepared from four groups of cells (same grouping as **A**, the raw figures in Supplementary Fig. 3C). **E** Transcriptional activity of the NR5A1 mutant. Dual-luciferase activity detected in cells co-transfected with the WT plasmid and *AMH* reporter or the NR5A1-Mut plasmid and *AMH* reporter. The internal fluorescence reference was pRL-TK Renilla luciferase; t-test was applied for.^*^p < 0.05

What’s more, in vitro functional verification experiment on the novel c.64G > T (p.G22C) variation in *NR5A1* were conducted. 293 T cells (ScienCell, Carlsbad, CA, USA), Myc-NR5A1 WT (WT) and Myc-p.G22C (NR5A1-Mut) plasmids, were used to conduct the *NR5A1*-overexpression system. qRT-PCR analysis showed no difference in *NR5A1* mRNA levels between cells transfected with WT or NR5A1-Mut plasmids (Fig. 5A). Western blot analysis subsequently confirmed that the protein of both WT and NR5A1-Mut groups was expressed at the same size (~ 53 kDa) and nearly the same level (Fig. 5B, Supplementary Fig. 3A&B). Immunofluorescence results revealed that WT and NR5A1-Mut localized exclusively to the nucleus (Fig. 5C), suggesting that the G22C substitution did not affect NR5A1 subcellular distribution. Electrophoretic mobility shift assays (EMSAs) were performed to determine the DNA-binding properties of the mutant. The results showed that the G22C substitution decreased the DNA-binding affinity of NR5A1 (Fig. 5D, Supplementary Fig. 3C). To determine transcriptional activity, the dual-luciferase reporter assay was performed using NR5A1 responsive promoter fragments of the human AMH gene. As shown in Fig. 5E, transactivation activity of the p.G22C mutant was clearly impaired.

## Discussion and conclusions

Although the diagnosis of 46,XY DSD is rare, the impact on the life quality of the affected adolescents and their families is significant even devastating [12]. Physicians involved in 46,XY DSD diagnoses agree that more work is required in this area. In the previous study, both the classic and new approach in parallel for the diagnosis were successfully implemented in a Chinese infant with 46,XY DSD [18]. In the present study, we proceeded the approach for the diagnosis of partial gonadal dysgenesis in a Chinese adolescent with resulting from a c.64G > T (p.G22C) *NR5A1* variant. Advanced genetic detection is applied as a first line of investigation for a molecular diagnosis and helpful to the etiologic diagnosis of patients with DSD and progressively improves the etiologic diagnostic rate [5, 7]. Following molecular diagnosis, further investigations are warranted to determine a more accurate phenotype and minimize unnecessary testing, sampling, and analysis. Molecular diagnosis allows a more reasonable sex assignment/re-assignment process and often helps individuals and families to cope with uncertainty, potential stigma, and accusations [10, 19], which are of great significance for adolescents with 46,XY DSD and their families. The application of these steps in the present case resulted in satisfactory follow-up treatment of the adolescent patient.

The emergence of new genetic techniques strongly influences the rate of correct diagnoses and reduces diagnostic delay [20]. The previous studies revealed a diagnostic rate of pathogenic variants identified in 46,XY DSD of ~ 40–66% [21]. In the present study, we identified a *NR5A1* mutational site using TRS in a Chinese adolescent with 46,XY DSD. This novel c.64G > T (p.G22C) variant is a heterozygous variant, similar to most reported *NR5A1* variants [6, 22–24]; however, the patient presented only gonadal dysgenesis without adrenal insufficiency. Heterozygous variants constitute the overwhelming majority of *NR5A1* variants in human [6].

To date, the reported *NR5A1* variants include missense and small deletions and insertions [6]. The novel c.64G > T (p.G22C) variant reported in the present study is a missense variant, which reportedly accounts for 58% of *NR5A1* variants [6]. The p.G22C substitution is located in the DBD, which is one of three domains in NR5A1, and previous studies have reported this as the location for multiple variants in patients with 46,XY DSD [6, 22, 24–26]. A previous study identified a G35E substitution in the DBD region in a patient with 46,XY DSD presenting adrenal insufficiency along with moderately severe gonadal dysgenesis, indicating that this variation results in serious adverse effects on NR5A1 function [22]. Additionally, the p.V15M, p.M78I, and p.G91S variations in this region are reportedly responsible for aberrant *NR5A1* transcription, with the first two variations resulting in altered subcellular localization [23]. Moreover, functional studies have shown that the p.S32N, p.N44del, and p.G91D variations in this region reduce the transactivation of cytochrome P450 family 11 subfamily A member 1 [25]. In the present study, functional analyses suggested that the p.G22C variant demonstrated a decreased ability to bind DNA, resulting in significantly reduced levels of *AMH* transcription.

Interestingly, the site of the amino acid substitution reported here (G22C) is the same as that described by Sudhakar et al. [27] (G22S); however, the phenotype of the two patients is completely different. The patient (G22S) has very small penis, penoscrotal hypospadias, and hypoplastic scrotum with reduced rugosity at age of 9 years old, with less failure degree of male sexual characteristics development than the current patient (G22C). These findings reinforce the difficulty associated with establishing a concise phenotype–genotype correlation in 46,XY DSD diagnoses.

Furthermore, although previous studies suggest that some NR5A1 variants alter the 3D structure of the protein [25, 28], we found that the p.G22C substitution has no effect on NR5A1 structure. However, subsequent functional verification suggested this site as pathogenic, indicating the need for further mechanistic studies.

AMH secreted by Sertoli cells immediately after testicular differentiation, is responsible for the regression of Müllerian ducts in the male fetus [29]. The state of the Müllerian derivatives reflects the effect of AMH secreted very early in fetal life [29]. The absent Müllerian derivatives in this patient indicated that AMH still perfectly performs the responsibility for the regression of Müllerian ducts, even though *NR5A1* variant may affect its function of promoting the secretion of AMH. In the human fetus at 9 weeks, Müllerian ducts have nearly totally disappeared [29]. It suggested that AMH was normal at least before 9 fetal weeks. The female external genitalia and dysplasia testicular tissue may be due to impaired androgens or androgen receptor secretion or action.

This patient was diagnosed with 46,XY DSD during adolescent years. The foreign and sudden disorders is undoubtedly an alarming and traumatic event for the adolescent and families. They have to face with some problems, including impaired fertility, medical treatment and possibly gonadal or vaginal surgery [3]. Sexuality is a sensitive topic for most Chinese families, and parents often avoid talking about it with children. The adolescent and parents experienced these painful sufferings. With the support and help of professionals in MDT of our hospital, they had slowly accepted all. It is only one case, however, we will continue to pay attention to DSD adolescents and look forward to sharing more with you in the future.

In summary, we described the diagnostic process for a 13-year-old Chinese patient with partial gonadal dysgenesis, including clinical and molecular diagnoses. Functional analysis identified a novel c.64G > T (p.G22C) variant in *NR5A1* as a pathogenic variant that resulted in the *NR5A1*-mediated dysregulation of gonadal development. This study adds one more *NR5A1* variant to the long list of previously published data for this gene and enriches the adolescents of information available about the *NR5A1* mutation spectrum in Chinese population. Genetic analysis of more samples of *NR5A1* variants and functional studies would be of great significance for understanding the mechanism of gonadal development and sex determination.

## Supplementary Information

**Additional file 1: Supplementary Fig 1.** Timeline of the diagnostic process for the patient.

**Additional file 2: Supplementary Fig 2.** Conformational changes in the NR5A1 mutant. The residual at position 22 changes from a hydrophobic to hydrophilic amino acid with the G22C substitution; both the WT and NR5A1-Mut proteins show the formation of a hydrogen bond with Thr29.

**Additional file 3: Supplementary Fig 3.** The raw figures of gels (EMSA) and the blots (Western blot) (A) the bands of the internal reference (β-actin). From left to right, these bands are non-transfected (Control, undeveloped band), transfected with an empty vector (Vector), Myc-tagged WT (WT), and c.64G>T (p. G22C) NR5A1-Mut (Mut) vectors; (B) NR5A1 expression in 293T cells according to western blot analysis. (same grouping as A); (C) ESMAs results showing altered DNA binding by the NR5A1 mutant (same group as A).

**Additional file 4: Supplementary Table 1.** List of 360 endocrine-related genes detected by TRS.

**Additional file 5: Supplementary Table 2.** Prediction of the impact of the c.64G>T (p.G22C) *NR5A1 *variant.

## Data Availability

The gene sequencing data of NR5A1 is stored in NCBI Sequence Read Archive (SRR22534035).

## Abbreviations

ACTH: Adrenocorticotropic hormone
AMH: Anti-Müllerian hormone
CNV-seq: Copy number variation sequencing
DBD: DNA-binding domain
DSD: Disorders/Differences of sex development
E2: Estradiol
EMSAs: Electrophoretic mobility shift assays
ERT: Estrogen-replacement therapy
FSH: Follicle-stimulating hormone
HCG: Human chorionic gonadotropin
HMG: Human menopausal gonadotropin
LH: Luteinizing hormone
NGS: Next-generation sequencing
NR5A1: Nuclear receptor subfamily 5 group A member 1
17-OHP: 17-Hydroxyprogesterone
SRY: Sex-determining region Y
T: Testosterone
TRS: Targeting specific disease‐causing genes
WT: Wild type
