# Supplementary figures and images for: A novel c.64G > T (p.G22C) NR5A1 variant in a Chinese adolescent with 46,XY disorders of sex development: a case report
Source: BMC Pediatr. 2023 Apr 19;23:182. doi: 10.1186/s12887-023-03974-7 (PMC10114376; doi:10.1186/s12887-023-03974-7)

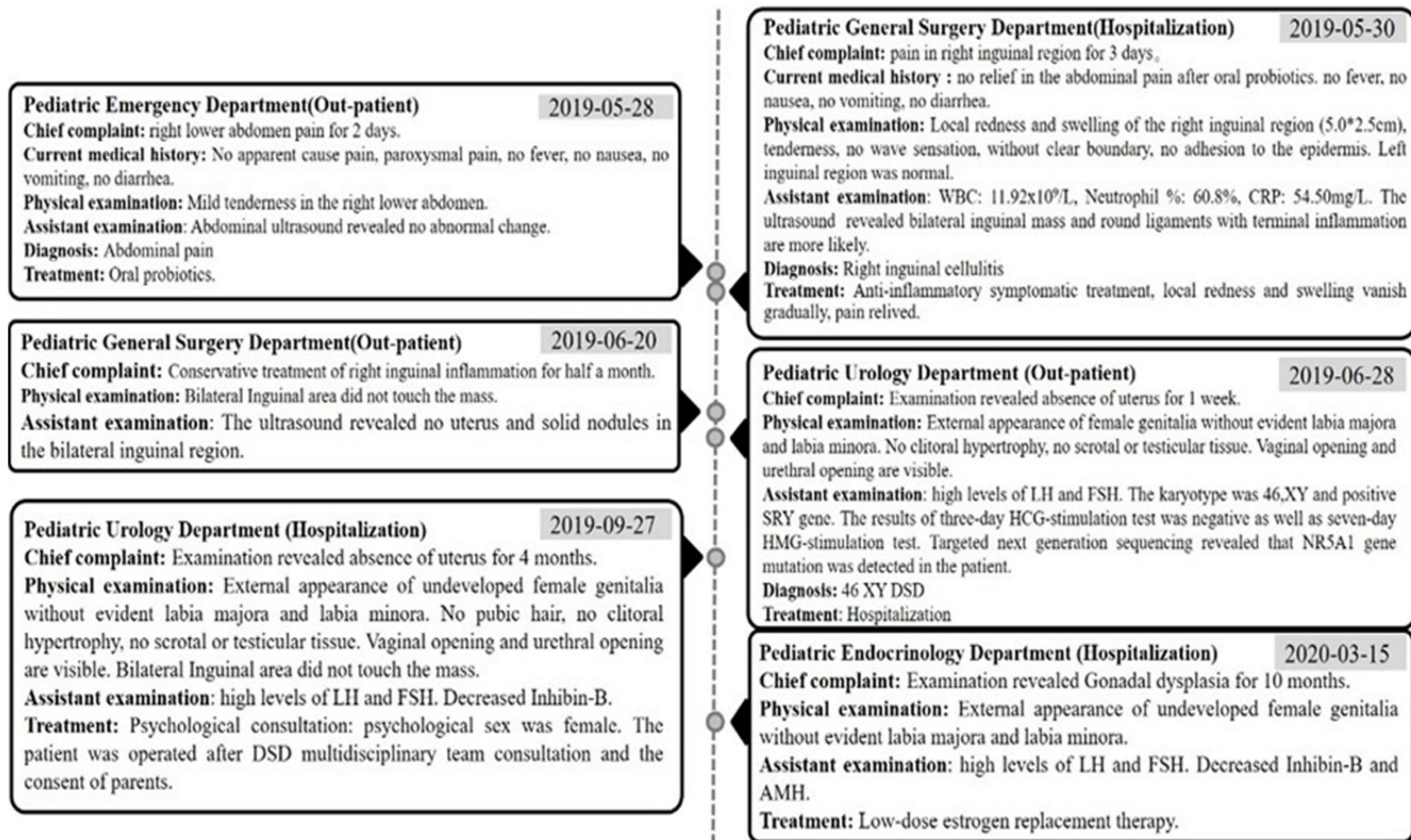

**Supplementary Fig. 1. Timeline of the diagnostic process for the patient.**

Supplement: Supplementary file 1 — Additional file 1: Supplementary Fig 1. Timeline of the diagnostic process for the patient. [file 12887_2023_3974_MOESM1_ESM.pdf]
