# Supplementary material for: A novel c.64G > T (p.G22C) NR5A1 variant in a Chinese adolescent with 46,XY disorders of sex development: a case report
Source: BMC Pediatr. 2023 Apr 19;23:182. doi: 10.1186/s12887-023-03974-7 (PMC10114376; doi:10.1186/s12887-023-03974-7)
Supplement: Supplementary file 2 — Additional file 2: Supplementary Fig 2. Conformational changes in the NR5A1 mutant. The residual at position 22 changes from a hydrophobic to hydrophilic amino acid with the G22C substitution; both the WT and NR5A1-Mut proteins show the formation of a hydrogen bond with Thr29. [file 12887_2023_3974_MOESM2_ESM.pdf]

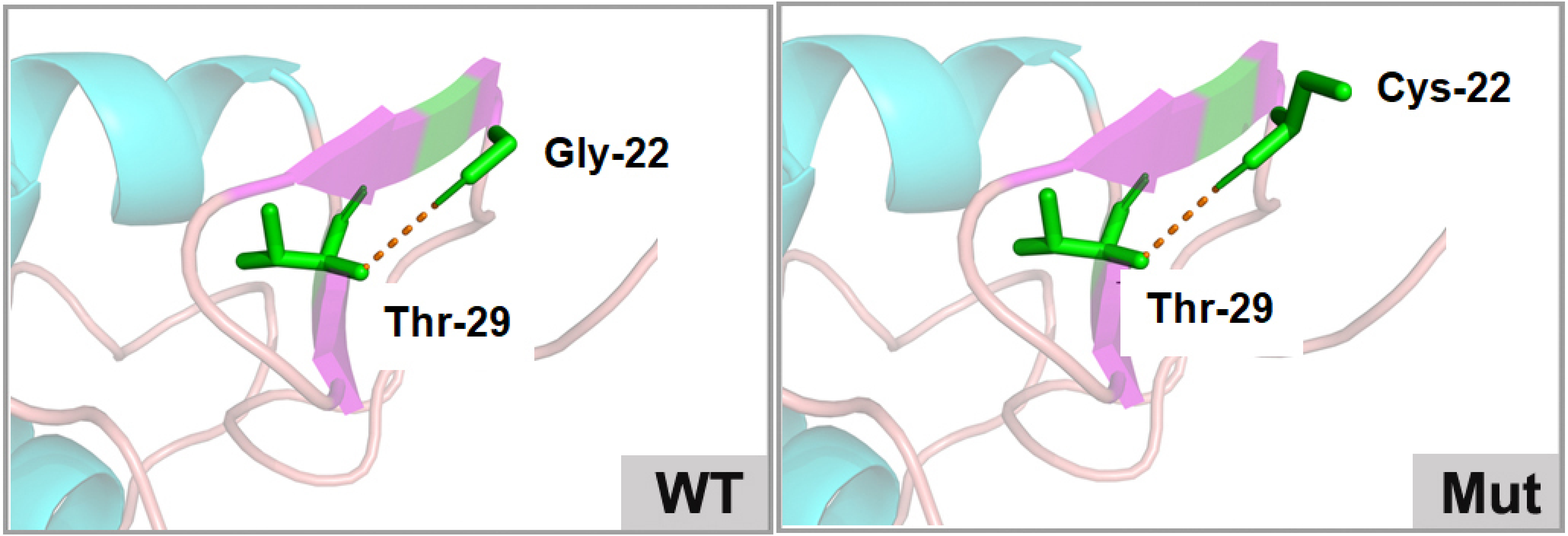

Supplementary Fig. 2. Conformational changes in the NR5A1 mutant. The residue at position 22 changes from a hydrophobic to hydrophilic amino acid with the G22C substitution; both the WT and NR5A1-Mut proteins show the formation of a hydrogen bond with Thr29.
