# Supplementary material for: A novel c.64G > T (p.G22C) NR5A1 variant in a Chinese adolescent with 46,XY disorders of sex development: a case report
Source: BMC Pediatr. 2023 Apr 19;23:182. doi: 10.1186/s12887-023-03974-7 (PMC10114376; doi:10.1186/s12887-023-03974-7)
Supplement: Supplementary file 3 — Additional file 3: Supplementary Fig 3. The raw figures of gels (EMSA) and the blots (Western blot) (A) the bands of the internal reference (β-actin). From left to right, these bands are non-transfected (Control, undeveloped band), transfected with an empty vector (Vector), Myc-tagged WT (WT), and c.64G>T (p. G22C) NR5A1-Mut (Mut) vectors; (B) NR5A1 expression in 293T cells according to western blot analysis. (same grouping as A); (C) ESMAs results showing altered DNA binding by the NR5A1 mutant (same group as A). [file 12887_2023_3974_MOESM3_ESM.pdf]

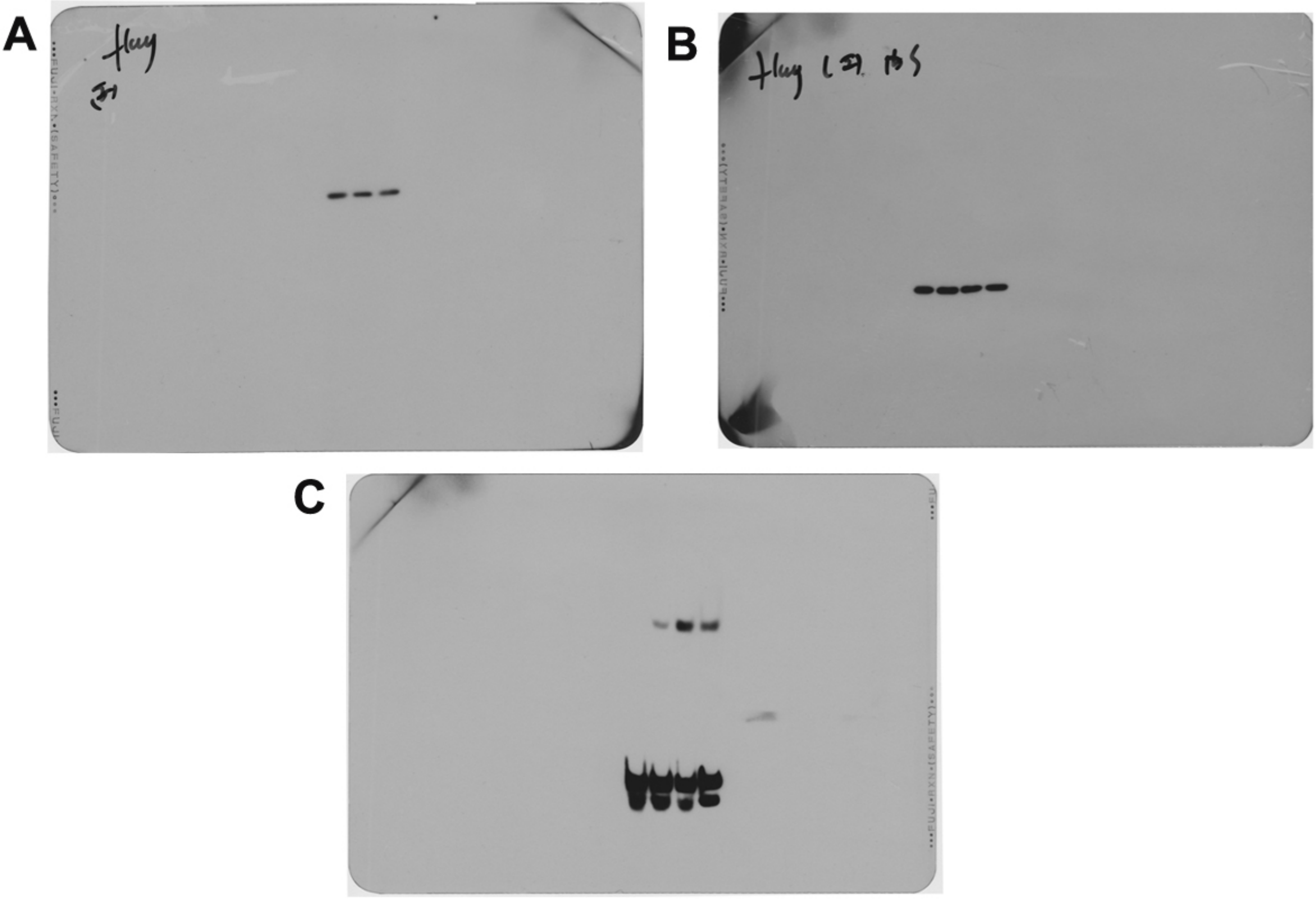

Supplementary Fig. 3. The raw figures of gels (EMSA) and the blots (Western blot) (A) The bands of the internal reference (β-actin). From left to right, these bands are non-transfected (Control,undeveloped band) , transfected with an empty vector (Vector), Myc-tagged WT (WT), and c.64G>T (p.G22C) NR5A1-Mut (Mut) vectors; (**B**) NR5A1 expression in 293T cells according to western blot analysis. (same grouping as A) ; (**C**) EMSAs results showing altered DNA binding by the NR5A1 mutant (same grouping as **A**).
