# Supplementary material for: A novel c.64G > T (p.G22C) NR5A1 variant in a Chinese adolescent with 46,XY disorders of sex development: a case report
Source: BMC Pediatr. 2023 Apr 19;23:182. doi: 10.1186/s12887-023-03974-7 (PMC10114376; doi:10.1186/s12887-023-03974-7)
Supplement: Supplementary file 4 — Additional file 4: Supplementary Table 1. List of 360 endocrine-related genes detected by TRS. [file 12887_2023_3974_MOESM4_ESM.pdf]

Supplementary Table 1. List of 360 endocrine-related genes detected by TRS.

A2ML1	*CA2*	*DUOXA2*	*HARS2*	*LARS2*	*OTX2*	*ROR2*	*TACR3*	*ORC1*
AARS2	*CACNA1S*	*DUSP6*	*HDAC8*	*LHB*	*PALB2*	*RSPO1*	*TAF4B*	*RNF216*
ABCA1	*CASR*	*EIF2AK3*	*HESX1*	*LHCGR*	*PALLD*	*RXFP2*	*TBCE*	*TAC3*
ABCD1	*CBX2*	*EPO*	*HFM1*	*LHX4*	*PAX6*	*RXRB*	*TG*	*STAT5B*
ABCC8	*CATSPER1*	*ENPP1*	*HFE*	*LHX3*	*PAX4*	*RXRA*	*TBX19*	*STK11*
ACVR1B	*CCDC28B*	*ERCC6*	*HGF*	*LZTFL1*	*PAX8*	*RYR1*	*THRA*	*SYCP3*
AGT	*CD96*	*ERCC8*	*HNF1A*	*MAMLD1*	*PCM1*	*SCN4A*	*THRB*	*RFX6*
AIP	*CDC73*	*ESR1*	*HNF1B*	*MAP2K1*	*PCNT*	*SCNN1A*	*TMEM67*	*RIPK4*
AIRE	*CDKN1C*	*FEZF1*	*HNF4A*	*MAP2K2*	*PCSK1*	*SCNN1B*	*TP53*	*RIT1*
AKR1C2	*CDKN2A*	*FGD1*	*HOXA13*	*MAP3K1*	*PDE11A*	*SCNN1G*	*TPO*	*NSDHL*
AKR1C4	*CEL*	*FGF17*	*HRAS*	*MAPK8IP1*	*PDE8B*	*SDCCAG8*	*TRH*	*NSMF*
AKT2	*CEP19*	*FGF23*	*HS6ST1*	*MC2R*	*PDX1*	*SDHB*	*TRHR*	*NTRK1*
ALB	*CEP290*	*FGF8*	*HSD11B1*	*MCM4*	*PHEX*	*SDHD*	*TRIM24*	*KLHL10*
ALG3	*CFTR*	*FGFR1*	*HSD11B2*	*MCM9*	*PHF6*	*SECISBP2*	*TRIM32*	*KMT2D*
AMH	*CHD7*	*FIGLA*	*HSD17B3*	*MED12*	*PLAGL1*	*SEMA3A*	*TRIM33*	*KRAS*
AMHR2	*CHRM3*	*FLRT3*	*HSD17B4*	*MEN1*	*PNLIP*	*SEMA3E*	*TSHB*	*GOPC*
ANOS1	*CLCN5*	*FMR1*	*HSD3B2*	*MID1*	*POF1B*	*SHOC2*	*TSHR*	*GPR101*
APOA1	*CLCNKA*	*FOXE1*	*IARS2*	*MINPP1*	*POLR3A*	*SLC12A1*	*TSPYL1*	*H6PD*
AQP2	*CLCNKB*	*FOXE2*	*ICK*	*MKKS*	*POLR3B*	*SLC12A3*	*TTC8*	*DMP1*
AR	*CLDN2*	*FOXP3*	*IER3IP1*	*MKRN3*	*POMC*	*SLC16A1*	*TTR*	*DMRT1*
ARL6	*CLEC7A*	*FSHR*	*IGF1*	*MKS1*	*PON1*	*SLC16A2*	*TWNK*	*DPY19L2*
ARMC5	*CLPP*	*GATA3*	*IGF1R*	*MNX1*	*POR*	*SLC19A2*	*TXNRD2*	*BRCA1*
ARX	*COX4I2*	*GATA4*	*IGF2*	*MRAP*	*POU1F1*	*SLC26A3*	*UCP2*	*BRCA2*
ATM	*CPA1*	*GATA6*	*IGFALS*	*MYH8*	*PRKACA*	*SLC26A4*	*UBR1*	*BSND*
ATP6V0A4	*CTRC*	*GCK*	*IGSF1*	*NAA10*	*PRKAR1A*	*SLC26A8*	*USP9Y*	*DUOX2*
ATP6V0B1	*CTSB*	*GCM2*	*IGSF10*	*NANOS1*	*PRKCA*	*SLC2A2*	*VDR*	*HADH*
ATRX	*CUL3*	*GH1*	*IL17RD*	*NCOA4*	*PROK2*	*SLC34A3*	*VEGFA*	*KRT8*
AURKC	*CYB5A*	*GH2*	*IL1RN*	*NDUFA13*	*PROKR2*	*SLC4A1*	*WDPCP*	*SRY*
AVP	*CYP11A1*	*GHR*	*INS*	*NEK8*	*PROP1*	*SLC4A4*	*WDR11*	*STAG3*
AVPR2	*CYP11B1*	*GHRH*	*INSL3*	*NEUROD1*	*PRSS1*	*SLC5A5*	*WNK1*	*STAR*
BBS1	*CYP11B2*	*GHRHR*	*INSR*	*NEUROG3*	*PSMC3IP*	*SOD2*	*WNK4*	*RBBP8*
BBS10	*CYP17A1*	*GHSR*	*IRF6*	*NKX2-1*	*PTEN*	*SOS1*	*WNT3*	*REN*
BBS12	*CYP19A1*	*GK*	*IYD*	*NKX2-2*	*PTF1A*	*SOX10*	*WNT4*	*RET*
BBS2	*CYP21A2*	*GK2*	*KCNJ1*	*NKX2-5*	*PTH*	*SOX3*	*WNT5A*	*NR3C2*
BBS4	*CYP27B1*	*GLCCI1*	*KCNJ10*	*NNT*	*PTPN11*	*SOX9*	*WT1*	*NR5A1*
BBS5	*CYP2R1*	*GLIS3*	*KCNJ11*	*NOBOX*	*RAB23*	*SPATA16*	*ZFAT*	*NRAS*
BBS7	*DAZL*	*GLUD1*	*KCNJ2*	*NPHP3*	*RAB3GAP2*	*SPINK1*	*ZFP57*	*KLHL3*
BBS9	*DCAF17*	*GNAI2*	*KDM6A*	*NR0B1*	*RAF1*	*SPRY4*	*ZMYND15*	*GOLGA5*
BCAM	*DHCR7*	*GNAS*	*KISS1*	*NR3C1*	*RASA2*	*SRD5A2*	*BTK*	*DICER1*
BLK	*DHH*	*GNRH1*	*KISS1R*	*BMP15*	*DIAPH2*	*GNRHR*	*KLF11*	*BRAF*
