# Supplementary material for: A novel c.64G > T (p.G22C) NR5A1 variant in a Chinese adolescent with 46,XY disorders of sex development: a case report
Source: BMC Pediatr. 2023 Apr 19;23:182. doi: 10.1186/s12887-023-03974-7 (PMC10114376; doi:10.1186/s12887-023-03974-7)
Supplement: Supplementary file 5 — Additional file 5: Supplementary Table 2. Prediction of the impact of the c.64G>T (p.G22C) NR5A1 variant. [file 12887_2023_3974_MOESM5_ESM.pdf]

Supplementary Table 2. Prediction of the impact of the c.64G>T (p.G22C) *NR5A1* variant.

Prediction software	Score/Grade	Harmful	Definition of "harmful"
**Scale-invariant feature transform (SIFT)**	0.03	yes	Score <0.05
**Protein Variation Effect Analyzer (PROVEAN)**	−5.652	yes	≤ threshold (−2.5)
**Polymorphism Phenotyping v2 (PolyPhen-2)**	1.000	yes	Score ~0.447–0.909: potentially harmful<br>Score ~0.910–1.000: very likely to be harmful
**Align-Grantham Variation/Grantham Deviation (Align-GVGD)**	C65	yes	~C0–C65: predicted pathogenicity grade from low to high
